# Gut Microbiomes of Marine Zooplankton: Consequences for Host Performance, the Biological Carbon Pump, and Prokaryote Biogeography

**DOI:** 10.1111/1462-2920.70271

**Published:** 2026-04-05

**Authors:** Albert Calbet

**Affiliations:** ^1^ Institut de Ciències del Mar, CSIC Barcelona Spain

**Keywords:** carbon pump, copepod, functional traits, microbiome, physiology, prokaryote, zooplankton

## Abstract

Marine zooplankton are a link between primary producers and higher trophic levels and play a pivotal role in organic matter export via diel vertical migration and faecal‐pellet production. Molecular surveys have revealed each individual as a holobiont hosting dense, taxonomically structured microbiomes in the gut, on the cuticle, and within feeding structures. These microbial partners expand dietary breadth through carbohydrate‐active enzymes, supply limiting vitamins, detoxify xenobiotics, and may buffer thermal and oxidative stress, thereby influencing host fitness and the fate of particulate organic carbon. Experimental studies show intact microbiomes often enhance growth or fecundity, with effects amplified under vitamin limitation or toxin exposure. In contrast, gut and pellet‐associated respiration can substantially reduce the carbon content of pellets within hours to days, depending on temperature and microbial composition. Vertical migrators also transport live bacteria and viruses below the thermocline, potentially seeding mesopelagic niches and affecting remineralization patterns. Despite these influences, zooplankton holobionts remain largely absent from biogeochemical models. This minireview synthesises current understanding of microbiome assembly and plasticity, their contributions to host performance and carbon export, and their role in microbial dispersal, underscoring the need to integrate holobiont traits into Earth‐system models to better predict ecosystem responses to warming and deoxygenation.

## Graphical abstract

Expected effects of zooplankton microbiota on the host and on the ecosystem. Stylized mesozooplankton holobiont (center) with arrows indicating respiration within the gut and export of faecal pellets and associated microbes. Orange bubbles summarise microbiome‐mediated consequences for host performance (digestive help, detoxification, vitamin provisioning, stress buffering). Blue bubbles indicate consequences for the biological carbon pump (pre‐pellet and pellet respiration, enhanced aggregation and ballasting). The green bubble represents consequences for prokaryote biogeography (vertical and horizontal transport of gut‐ and surface‐associated microbes). Together, these processes connect individual‐level holobiont traits to ecosystem‐scale carbon and nutrient cycling.

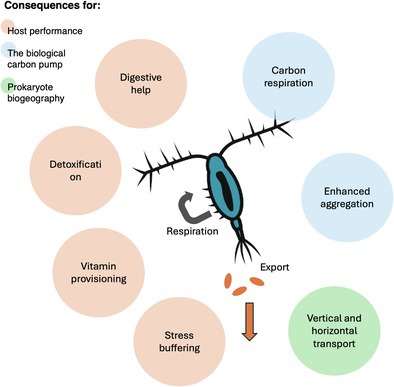

## 1 Introduction

Marine mesozooplankton comprise a major fraction of metazoan biomass in the ocean and mediate large shares of pelagic energy flow and biogeochemical cycling through grazing, respiration, excretion, vertical migration and the production and reworking of faecal pellets, moults and carcasses (Calbet 2001; Collins et al. 2015; Steinberg and Landry 2017; Cavan et al. 2017). By transforming primary production into fast‐sinking particles and by fragmenting and ‘gardening’ detritus, they set both the magnitude and composition of particle export from the euphotic zone and thereby modulate the efficiency of the biological carbon pump (Cavan et al. 2017; Darnis et al. 2024). Classical bioenergetic formulations treated zooplankton as physiologically self‐contained units and the gut lumen as a passive conduit for nutrient partitioning. Amplicon and metagenomic surveys show that copepods and other zooplankton host dense, structured microbiomes that differ from seawater and are enriched in Rhodobacteraceae, Flavobacteriaceae, Vibrionaceae and Alteromonadales, including nitrate‐respiring Gammaproteobacteria (Shoemaker and Moisander 2015; De Corte et al. 2018; Moisander et al. 2018; Velasquez et al. 2023). These holobionts influence biogeochemistry along two principal pathways. First, pre‐pellet respiration by gut bacteria and archaea can oxidise a sizeable fraction of freshly ingested carbon, decreasing pellet POC and altering export efficiency (Tang et al. 2011); second, diel vertical migration displaces metazoans—and their viable microbiota—hundreds of metres across steep thermal and redox gradients, transporting cells, enzymes, metabolites and viruses into mesopelagic habitats (Grossart et al. 2010; Frada and Vardi 2015). While manipulative experiments demonstrate that microbiome disruption can retard development and survival in some marine and freshwater zooplankton and that toxin‐degrading or vitamin‐producing bacteria can enhance reproduction under specific conditions, robust quantification of net fitness effects across temperature, oxygen, diet and contaminant gradients remains limited; similarly, direct, in situ tracking of labelled gut bacteria from epipelagic hosts to established deep‐water populations is still lacking (Fong et al. 2019; Gorokhova et al. 2021; Sadaiappan et al. 2021; Cooper et al. 2022; Carrillo et al. 2023; Vu et al. 2024). Here, I emphasise trait‐based generalities across taxa—rather than taxonomic identity per se—and integrate single‐organism ‘omics, microsensor profiling and process studies’ to outline the consequences of zooplankton holobionts for host life histories, the magnitude and efficiency of export and microbial biogeography. In the sections that follow, I first synthesise current knowledge of microbiome assembly and plasticity in marine zooplankton, with emphasis on copepods. I then examine how gut and surface consortia influence host performance through digestion, vitamin and toxin dynamics and stress buffering, before turning to their roles in modulating faecal‐pellet transformation, export efficiency and microbial dispersal via vertical migration. Finally, I highlight outstanding questions and outline experimental and observational strategies to quantify zooplankton holobiont contributions to regional carbon and nitrogen budgets.

## 2 Microbiome Assembly and Plasticity

Across habitats, zooplankton guts are assembled from the surrounding bacterial reservoir but are not random seawater subsets; instead, they are enriched in particle‐associated lineages with traits that favour adhesion, rapid growth, and survival in a transient, nutrient‐rich and often hypoxic lumen (Cnudde et al. 2013; Shoemaker and Moisander 2015; Feng et al. 2023). The same types of lineages are abundant on expelled faecal pellets that act as microbial microhabitats distinct from free‐living assemblages (Shoemaker and Moisander 2015; De Corte et al. 2017). Gut oxygen microprofiles collected with microelectrodes show that, following feeding, oxygen concentrations in the guts of *Calanus* spp. drop from near air saturation at the periphery to hypoxic or anoxic levels in the core, establishing radial gradients that select for facultative anaerobes and shift metabolic pathways toward mixed‐acid fermentation or alternative electron acceptors (Tang et al. 2011). Such gut‐scale redox structure provides a mechanistic filter consistent with the frequent detection of nitrate‐reductase gene potentials in copepod‐associated metagenomes (Moisander et al. 2018).

At larger scales, environmental forcing and feeding strategy are strong determinants of community composition. For instance, field surveys in warm, nutrient‐poor systems have shown that ambush or mixed‐feeding copepods host distinct microbiomes compared to strict filter feeders (Velasquez et al. 2023). Diet quality and toxins further modulate assembly: during Baltic cyanobacterial blooms, copepod gut communities shift toward microcystin/nodularin degraders (mlrA–D carriers) (Gorokhova et al. 2021). Contaminants impose additional selection, as shown by dissolved‐copper exposures that restructure copepod‐associated communities toward metal‐handling taxa and genes without parallel shifts in host transcriptomes (Colin et al. 2023). Phylosymbiosis signals exist but are generally weaker than environmental drivers; distinct mesopelagic animals in Monterey Canyon carry characteristic microbiomes, yet inter‐individual variability is high and core taxa are few, indicating lottery‐type assembly with functional redundancy (Breusing et al. 2022). Overall, even though the specific microbes living with zooplankton can change quickly, their roles appear more stable over time and across individuals, a pattern that still needs to be tested directly by tracking key traits under changing temperature, oxygen, toxic prey and diet regimes (Cnudde et al. 2013; Yang et al. 2024).

Comparative work across marine and freshwater systems also reveals substantial variability in how strongly different zooplankton taxa filter and regulate their microbiomes. In some crustacean zooplankton, gut communities are clearly distinct from surrounding water, whereas in others they closely mirror ambient particle‐associated assemblages, suggesting weaker host control (Shoemaker et al. 2017; Eckert et al. 2021). Field surveys and experiments in lakes show that zooplankton microbiomes are highly flexible and largely structured by environmental conditions and host feeding mode, with only a small proportion of persistent ‘core’ taxa within each species (Eckert et al. 2021). More recent work indicates that geography and host identity jointly shape bacterial communities associated with different copepod and cladoceran hosts, with bacterioplankton remaining relatively stable while zooplankton microbiomes vary more strongly over space and among host taxa (Wall et al. 2025). In pelagic tunicates such as *Dolioletta gegenbauri*, highly consistent microbiomes dominated by specific lineages point to tighter host regulation and a potential role as links between grazing and microbial food webs (Pereira et al. 2023). Together, these examples show that zooplankton range from relatively permissive to strongly filtering hosts, but the underlying regulatory mechanisms—immune effectors, gut pH and redox microgradients, mucus chemistry and transit times—are only beginning to be explored in marine species.

## 3 Microbiome Contributions to Zooplankton Performance

**FIGURE 1:**
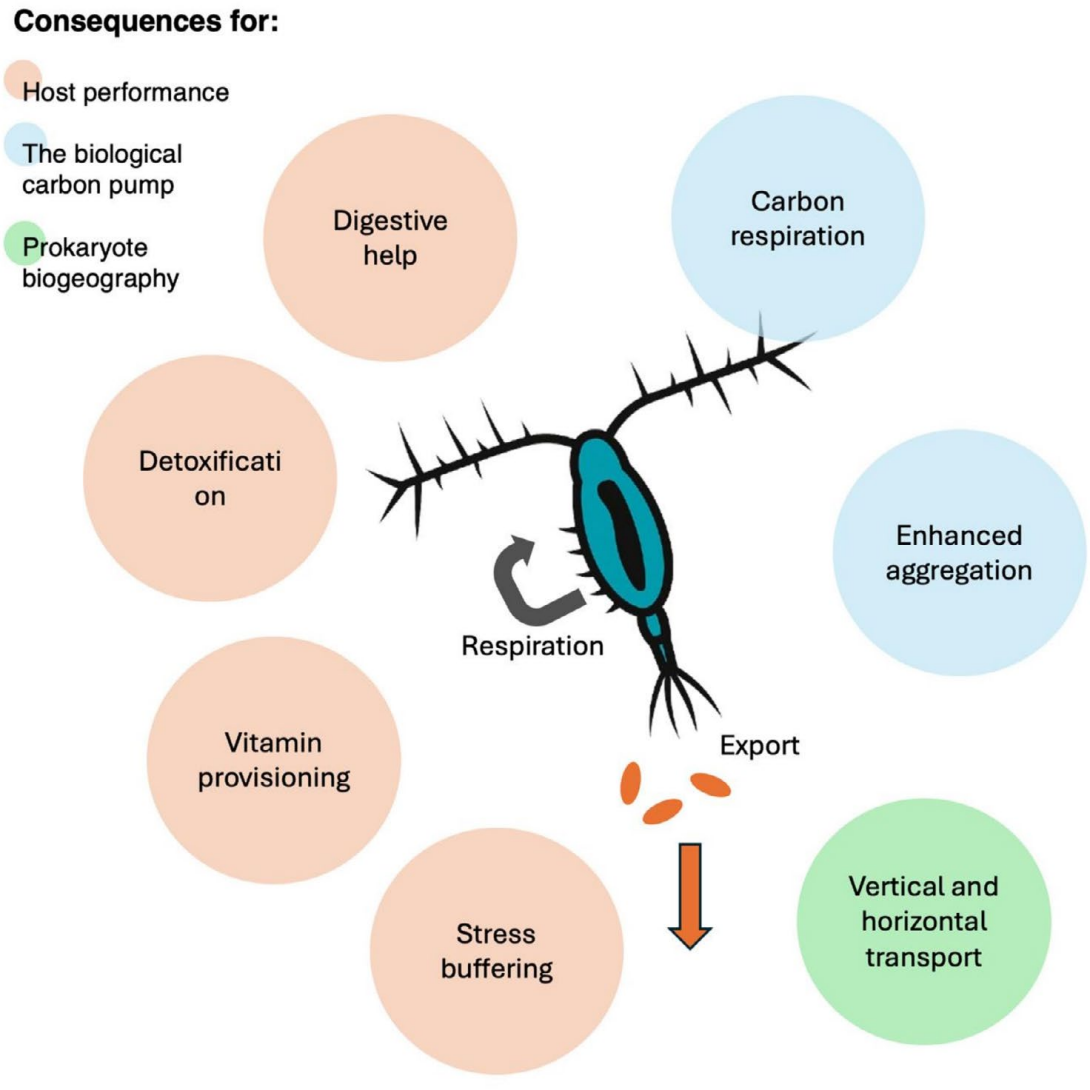
Expected effects of zooplankton microbiota on the host and on the ecosystem. Stylized mesozooplankton holobiont (center) with arrows indicating respiration within the gut and export of faecal pellets and associated microbes. Orange bubbles summarise microbiome‐mediated consequences for host performance. Blue bubbles indicate consequences for the biological carbon pump. The green bubble represents consequences for prokaryote biogeography.

Microbiome Services to Hosts Span Digestive Expansion, Vitamin Provisioning, Detoxification and Potential Stress Buffering (Figure 1).

Gut and faecal‐pellet communities express suites of carbohydrate‐active enzymes capable of cleaving chitin and refractory algal polysaccharides, thereby converting otherwise inaccessible carbon into assimilable substrates and stimulating pre‐pellet respiration (De Corte et al. 2017; Tang et al. 2011). Vitamin limitation is a widespread feature of planktonic systems: many microalgae are auxotrophic for cobalamin or thiamine and copepod‐associated microbiomes frequently harbour the corresponding biosynthetic potentials, consistent with a role in nutrient provisioning to the holobiont and its prey field (Croft et al. 2005; Sañudo‐Wilhelmy et al. 2014; Sadaiappan et al. 2021). Detoxification is now directly demonstrated in the field: experimental disruption of gut microbiomes with antibiotics during Baltic cyanobacterial blooms increases nodularin burdens and depresses hatching success in copepods, directly linking toxin‐degrading consortia to life‐history endpoints (Gorokhova et al. 2021). Yang et al. (2024) showed that copepod‐associated bacterial communities vary across geographic regions and seasons but maintain core functional traits, including capacities to cope with toxic prey, reinforcing the idea that host–microbe associations are selected for key functions rather than fixed taxonomic identities.

Experimental studies also show that disrupting copepod microbiomes with antibiotics can severely impair host performance, including complete developmental arrest in *Nitocra spinipes* exposed to trimethoprim (Edlund et al. 2012) and that temperature‐driven changes in microbiome composition are associated with marked shifts in development rate, growth, fecundity, gut diversity and the prevalence of oxidative‐stress genes at thermal extremes (Vu et al. 2024). Together, these findings support—but do not yet fully prove—causal contributions of the microbiome to digestive efficiency, toxin handling, and thermal tolerance.

## 4 Microbiome‐Mediated Modulation of the Biological Carbon Pump

Microbial activity in and on zooplankton alters both how much carbon leaves the surface ocean and what happens to that carbon en route to depth (Figure 1). Inside guts, rapid deoxygenation and steep redox gradients mean that a substantial fraction of ingested carbon can be respired or fermented before it is packaged into faecal pellets; after egestion, pellet‐attached bacteria continue to hydrolyse polymers using chitinases, proteases, β‐glucosidases, lipases, and other enzymes, often reducing pellet particulate organic carbon (POC) by 30%–50% over hours to days, depending on temperature, diet, and community composition (Tang et al. 2011; Cavan et al. 2017). In this context, ‘erosion’ refers to the enzymatic conversion of pellet POC back into dissolved or fine‐particulate forms that can be taken up by free‐living bacteria. This erosion channels carbon into upper‐mesopelagic microbial loops, tightening the recycling of organic matter near the base of the euphotic zone.

At the same time, zooplankton can increase the amount of material available for export by producing abundant pellets and by facilitating the aggregation and ballasting of organic matter. Mineral ballast (e.g., biogenic calcium carbonate or opal) increases particle density and settling speed, whereas transparent exopolymer particles (TEP) and other gels enhance particle stickiness and collision efficiency; the net effect on export depends on the balance between densification and microbial erosion (Passow and De La Rocha 2006; Engel 2020; Cavan et al. 2017). At the scale of budgets and models, explicitly representing zooplankton‐mediated respiration and pellet transformation generally reduces export relative to idealised ‘sterile‐zooplankton’ assumptions and can yield closer agreement with tracer‐based flux estimates, but the magnitude of this reduction varies strongly with region, food‐web structure and parameterization (Henson et al. 2019, 2022). As the ocean warms and oxygen minima shoal, shifts in community size spectra, feeding modes and food‐web routing will alter both microbe‐mediated attenuation and pellet production; constraining the temperature, oxygen and substrate sensitivities of gut and pellet processes is therefore a priority for the next generation of trait‐explicit biological pump models (Henson et al. 2024; Iversen 2023).

Although this review emphasises carbon, many of the same microsites are also hotspots of nitrogen transformations. Copepods host diazotrophic consortia, including unicellular cyanobacteria and non‐cyanobacterial diazotrophs, with measurable N_2_‐fixation rates at the individual level (Scavotto et al. 2015). Sinking carcasses and faecal aggregates foster anaerobic pathways such as dissimilatory nitrate reduction to ammonium, denitrification and anammox, contributing to fixed‐nitrogen loss at oxygen‐minimum‐zone boundaries (Stief et al. 2017; Bianchi et al. 2018). These nitrogen‐cycle processes feed back on carbon export because they regulate nutrient recycling, the degree of nitrogen limitation in surface waters and the stoichiometry of sinking material. A fuller integration of zooplankton‐associated nitrogen cycling with carbon‐focused particle models is therefore a logical next step.

## 5 Vertical Migration as a Vector for Microbial Biogeography

The dusk‐to‐dawn displacement of vast zooplankton biomass constitutes the largest daily animal migration on Earth and has the potential to redistribute microorganisms and their genes vertically (Figure 1). Zooplankton disperse bacteria both as hitchhikers on exoskeletons and via faecal strings that transport gut‐selected consortia below the thermocline; trap and incubation studies show that pellet‐attached communities are compositionally distinct from the surrounding deep‐water microbial assemblages and can remain viable for hours to days under high pressure, indicating a plausible contribution to mesopelagic particle‐associated niches rather than to free‐living deep assemblages (Grossart et al. 2010; De Corte et al. 2017). Viruses can also hitchhike on zooplankton: during *Emiliania huxleyi* blooms, grazing transfers coccolithoviruses between hosts, potentially accelerating bloom termination and revealing a zooplankton–virus conduit with ecosystem‐scale consequences (Frada et al. 2014).

While zooplankton‐mediated paths (vertical migration, faecal pellet production) may deliver significant fractions of viable cells below the thermocline, the long‐term fate of arrivals and their contribution to deep‐water community assembly remain to be quantified with pressure‐retaining, isotopically labelled tracers and coordinated depth‐stratified sampling.

## 6 Discussion and Outlook

The accumulating molecular, physiological and modelling evidence positions zooplankton holobionts as mobile, microbially augmented biogeochemical reactors whose internal processes resonate from individual performance to basin‐scale carbon sequestration. Microbiome functions—nutrient provisioning, detoxification, stress buffering and expanded digestive chemistry—can measurably enhance growth and fecundity under the right environmental and dietary contexts (Edlund et al. 2012; Gorokhova et al. 2021; Vu et al. 2024), whereas gut and pellet‐attached respiration and enzymatic erosion attenuate particle carbon and feed upper‐mesopelagic microbial loops (Tang et al. 2011; Cavan et al. 2017). Net effects on export efficiency, therefore, hinge on regional balances among temperature, oxygen, host size and feeding mode, diet composition and microbial traits, arguing for trait‐explicit representations of holobiont processes in Earth‐system models (Bianchi et al. 2018; Henson et al. 2019, 2022). Quantitative partitioning of host versus microbiome carbon and nutrient flows across temperature and oxygen gradients has been directly measured only in a handful of species and contexts—for example, gut microelectrodes in *Calanus* spp. and a few pellet‐erosion case studies (Tang et al. 2011; Cavan et al. 2017)—while genomic and transcriptomic evidence for anaerobic respiration in copepod‐associated bacteria remains largely unscaled to host–microbe budgets (Moisander et al. 2018; De Corte et al. 2017). Recent syntheses and modelling reviews repeatedly flag the absence of such partitioned measurements—separating host and microbial contributions—as a key bottleneck for predictive skill (Kelly et al. 2019; Iversen 2023; Henson et al. 2024). Linking gut and pellet microelectrode measurements with nanoSIMS imaging and single‐organism ‘omics along latitudinal transects would allow us to assign respiration, fermentation and nutrient‐transformation fluxes to hosts and microbes at cellular resolution and then scale those fluxes up along environmental gradients. In parallel, trait surveys that combine high‐throughput culturing of key bacterial lineages with metagenomic screening are needed to derive robust temperature, pressure and redox sensitivity functions for vitamin synthesis, toxin degradation and anaerobic respiration pathways (Fang et al. 2010; Wienhausen et al. 2022).

**FIGURE 2:**
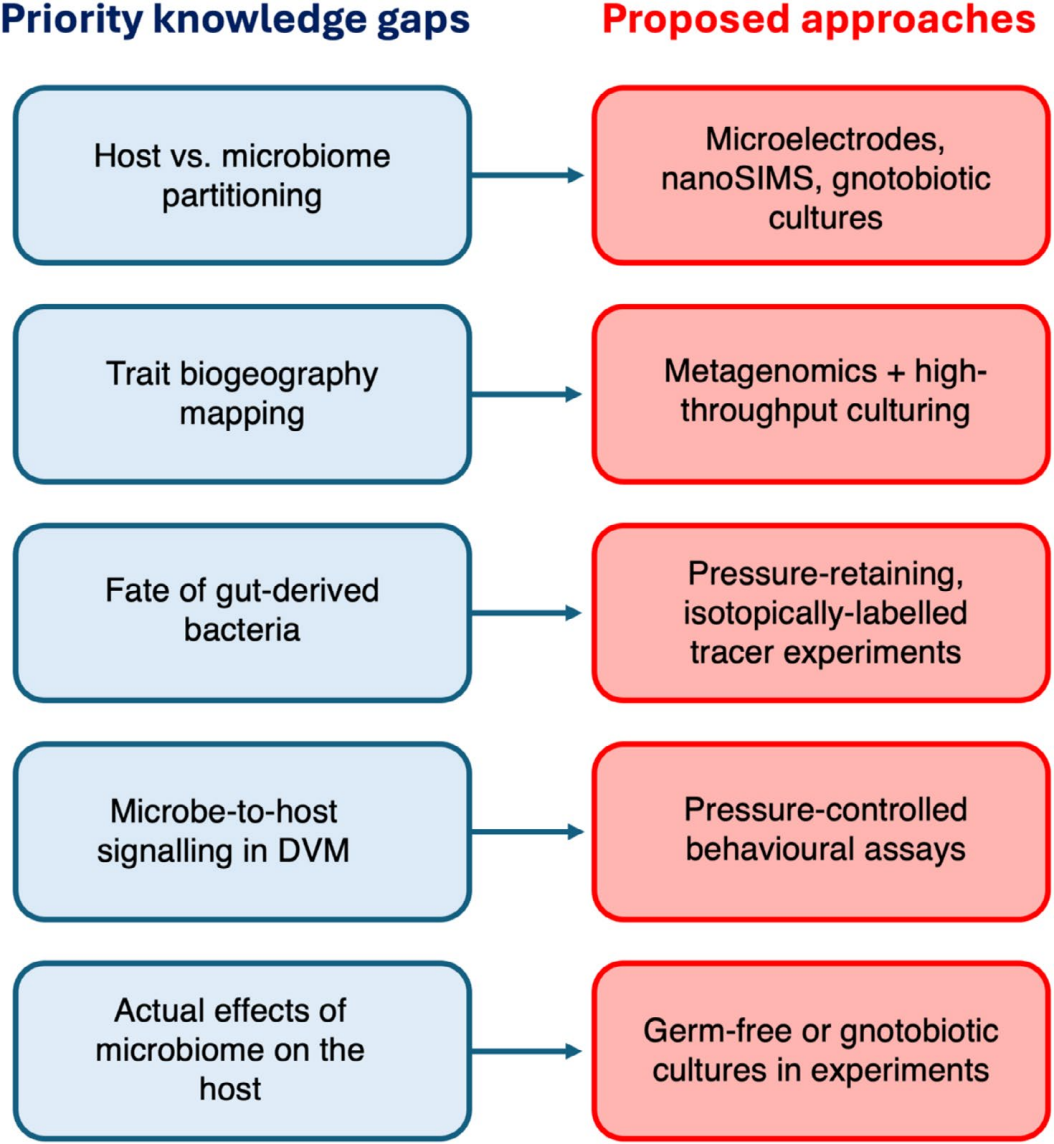
Unresolved issues in zooplankton holobiont research and proposed approaches. Blue boxes summarise five priority knowledge gaps: (i) quantitative partitioning of host versus microbiome metabolism, (ii) mapping the biogeography of key microbial traits, (iii) tracking the fate of gut‐derived bacteria exported below the thermocline, (iv) testing hypothesized microbe‐to‐host signalling effects on diel vertical migration; and (v) quantifying the net effects of microbiomes on host growth, survival, and behaviour. Arrows point to methodological approaches best suited to each problem.

The survival and colonisation fate of gut‐derived bacteria delivered below 1 km have likewise not been followed with pressure‐retaining, isotopically labelled tracers; such experiments would calibrate decay constants and colonisation probabilities for model parameterization and clarify to what extent diel vertical migration acts as a microbial ‘conveyor belt’ versus a short‐lived inoculation mechanism. Hypothesized microbe‐to‐host signalling effects on diel vertical migration also remain untested in ecologically dominant taxa under realistic hydrostatic, thermal and oxygen regimes. Addressing these questions will require coordinated field programmes that integrate behavioural assays with manipulations of microbiome composition, for example through germ‐free or gnotobiotic hosts reared under controlled pressure and temperature. Figure 2 distils these knowledge gaps into five priority problems paired with methodological approaches best suited to each.

Projected changes in phytoplankton communities and primary production will likely reverberate through zooplankton holobionts. Recent CO_2_‐enrichment experiments in the western North Pacific indicate that small (< 20 μm) eukaryotic phytoplankton, which are often nitrogen limited, drive consistent declines in community primary production under elevated *p*CO_2_, whereas prokaryotic phytoplankton show neutral or positive responses (Dai et al. 2025). Satellite‐based reconstructions further suggest that net primary production has already declined significantly across large expanses of the tropical and subtropical stratified ocean, largely due to strengthening nutrient limitation (Silsbe et al. 2025). Such shifts in the quantity and quality of phytoplankton prey will alter dietary substrates, toxin exposure and vitamin supply to zooplankton and their microbiomes, potentially reshaping holobiont trait distributions. Work on vitamin auxotrophy and toxin handling shows that B‐vitamin supply and cyanotoxin degradation by bacteria are key determinants of phytoplankton composition and copepod performance (Croft et al. 2005; Sañudo‐Wilhelmy et al. 2014; Gorokhova et al. 2021; Yang et al. 2024). In parallel, studies in lakes demonstrate that cyanobacterial dominance is closely linked to dissolved organic nitrogen and altered dissolved organic matter quality (Recknagel et al. 2022), implying changes in the substrates available to both free‐living and host‐associated bacteria. However, direct evidence that climate‐driven phytoplankton shifts reconfigure zooplankton gut microbiomes in the ocean is still scarce and testing these links should be a priority for future field studies and model development.

A further unresolved issue is how environmental stressors interact with microbiome composition and function to shape zooplankton performance. Temperature anomalies, pollutant exposure, hypoxia and food deprivation each alter host physiology directly, but they also restructure gut and surface microbial communities (Callens et al. 2015; Colin et al. 2023; Sison‐Mangus et al. 2015). These compositional changes can either exacerbate stress—for example, by reducing vitamin provision or increasing pathogen load—or buffer it via detoxification and nutrient supplementation (Gorokhova et al. 2020, 2021). Yet in most manipulative studies to date, environmental stressors simultaneously alter host physiology and microbiome composition, so that changes in growth or reproduction cannot be uniquely attributed to one side of the holobiont. Disentangling these pathways requires experimental designs that decouple host and microbiome responses—for example, germ‐free or gnotobiotic hosts that can be inoculated with defined microbial consortia under factorial combinations of temperature, oxygen and diet. Without this resolution, predictive models risk conflating host intrinsic tolerance with holobiont‐mediated resilience or vulnerability.

Overall, recognising zooplankton as holobiont actors rather than sterile carbon conduits is not merely rhetorical; it provides the mechanistic basis for developing trait‐based parameterizations of gut respiration, pellet transformation and microbial dispersal that can be incorporated, at appropriate levels of complexity, into regional and global biogeochemical models.

## Author Contributions

A.C. conducted the review and wrote the manuscript.

## Funding

This work was supported by MICIU/AEI (PID2023‐150548NB‐I00), ERDF/EU, and Consejo Superior de Investigaciones Científicas.

## Conflicts of Interest

The author declares no conflicts of interest.

## Data Availability

Data sharing not applicable to this article as no datasets were generated or analysed during the current study.
